# Cell invasion, RAGE expression, and inflammation in oral squamous cell carcinoma (OSCC) cells exposed to e‐cigarette flavoring

**DOI:** 10.1002/cre2.314

**Published:** 2020-08-11

**Authors:** Kary Y.F. Tsai, Kelsey M. Hirschi Budge, Anthony P. Lepre, Michael S. Rhees, Janet Ajdaharian, Jordy Geiler, Daniel G. Epperson, Kolten J. Astle, Duane R. Winden, Juan A. Arroyo, Paul R. Reynolds

**Affiliations:** ^1^ Lung and Placenta Research Laboratory, Department of Physiology and Developmental Biology Brigham Young University Provo Utah USA; ^2^ College of Dental Medicine, Roseman University of Health Sciences South Jordan Utah USA

**Keywords:** carcinoma, eCig, oral, RAGE

## Abstract

**Objective:**

Electronic cigarettes have given rise to a new, largely unregulated market within the smoking industry. While generally supposed to be less harmful than traditional tobacco smoke, awareness of the biological effects of electronic cigarette liquid is still scarce. Our objective was to determine the impact of electronic cigarette flavoring and nicotine on gingival squamous cell carcinoma invasion, RAGE expression, and the elaboration of pro‐inflammatory molecules.

**Methods and Materials:**

Gingival and tongue squamous cell carcinoma cells were exposed to Red Hot or Green Apple flavored electronic cigarette flavoring with or without nicotine. Immunofluorescence determined RAGE expression. Real‐time cellular invasion was assessed using a RTCA DP instrument. Culture medium was assayed for cytokine secretion.

**Results:**

Compared to controls we observed: increased cell invasion in gingival cells with Red Hot electronic cigarette flavoring and decreased cell invasion with Green Apple; decreased cell invasion in tongue cells treated with Red Hot electronic cigarette flavoring and no differences in invasion with Green Apple; flavor and nicotine dependent increases in RAGE expression; and differential expression of IL‐1α, IL‐8, and MMP‐13.

**Conclusion:**

We conclude that electronic cigarette flavoring and nicotine orchestrate differential regulation of oral squamous cell carcinoma (OSCC) cell invasion and inflammatory effects. This study provides an important initial step in dissecting RAGE‐mediated mechanisms of cancerous invasion and molecular avenues employed by OSCC.

## 1 INTRODUCTION

Electronic Cigarette (eCig) use has increased dramatically during the last several years. Numerous reports have sought to establish these products as safer to use than conventional cigarettes (Pisinger, Godtfredsen, & Bender, 2019; Pisinger & Mackay, 2019). The increased use is further attributed to the notion that eCig prevents the adverse effects of combustible cigarettes and may be pursued as a means that assists smokers in their cessation efforts (Alzahrani, Pena, Temesgen, & Glantz, 2018). eCigs are hand‐held electronic devices that generate vapors or aerosols from e‐cigarette liquid (eCig liquid) without combustion (Chen, Todd, & Fairclough, 2019). eCig liquid usually contains a mixture of propylene glycol, glycerin, nicotine, and flavors (Alzahrani et al., 2018; Uchiyama et al., 2016). Although they are increasing in their popularity, the World Health organization (WHO) regards the use of eCigs as harmful and does not recommend their ongoing use (Chen et al., 2019; Pisinger et al., 2019; Pisinger & Mackay, 2019). Previous experimental results showed that eCigs with flavorings caused increased oxidative/carbonyl stress, increased inflammatory cytokine release, increase receptor for advanced glycation end products (RAGE) expression, and increased apoptosis in human gingival cells (Rouabhia et al., 2017; Sundar, Javed, Romanos, & Rahman, 2016).

The RAGE is a pattern‐recognition cell‐surface receptor that experiences increased expression during exposure to tobacco smoke (Chapman et al., 2018; Robinson, Stogsdill, Lewis, Wood, & Reynolds, 2012; Winden et al., 2014). Expression of RAGE has been detected in gingival tissues from human subjects with chronic periodontitis and it is overexpressed in gingival tissues from smokers diagnosed with periodontal diseases (Chapman et al., 2018). RAGE receptor availability is implicated in the pathogenesis of many inflammatory diseases and more recently, it is been shown to be involved in the invasion of oral squamous cell carcinoma (OSCC) (Bhawal et al., 2005; Chapman et al., 2018).

In the current research endeavor, we examined the effects of two common eCig flavors, sweet apple and cinnamaldehyde, with or without nicotine, on OSCC invasiveness and the effect of exposure on RAGE expression. We also tested the inflammatory profiles of exposure via the assessment of elaborated mediators into cell culture media. OSCC encompasses up to 90% of all oral cancers in the United States and affects more than 400,000 individuals yearly (Chapman et al., 2018). OSCC is one of the most prevalent cancers in developing countries with recurrence rates that approach approximately 50%, and its progression is affected by environmental factors including cigarette smoke and alcohol (Bavle, Venugopal, Konda, Muniswamappa, & Makarla, 2016; Blatt et al., 2017). Tobacco alone is well recognized as one of the major causes of the development of oral cancer (Nagaraj & Zacharias, 2007) and work in our lab demonstrated that secondhand smoke increases OSCC invasion in a RAGE dependent manner (Chapman et al., 2018). The current project expands the invasive attributes of OSCC by seeking to test the hypothesis that eCig exposure mediates cellular invasion and inflammation possibly orchestrated by the induction of RAGE.

## 2 MATERIALS AND METHODS

### 2.1 Cell culture and treatments

Ca9‐22 oral squamous carcinoma cells and CAL‐27 human tongue squamous carcinoma cells were used in these experiments (both from ATTC, Manassas, VA). Cells were cultured in RPMI medium (Mediatech, Manassas, VA) supplemented with 10% fetal bovine serum (FBS) and 1% penicillin and streptomycin. Both cell lines at approximately 80% confluence were incubated for 24 hr in medium alone (control), medium supplemented with 2% Cinnamon Red Hots (Red Hot, 8Ohm1, Inc., Ogden, UT) or Reds Apple Juice (Green Apple, Daze Mfg., Los Angeles, CA) eCig liquid in the presence or absence of 6 mg of nicotine. These flavors are amongst the more popular varieties and a dose curve was pursued and a concentration of 2% for both types of eCig liquid exerted changes without affecting cell behavior (data not shown). The concentration of nicotine was selected to be 6 mg following pilot projects that utilized concentrations between 2 and 12 mg and in order to identify a midrange concentration in accordance with current trends of usage (Romberg et al., 2019). For invasion studies, cells were detached, and 20,000 cells/mL were incubated in 2% FBS medium alone, or medium supplemented with 2% eCig liquid in the presence or absence of nicotine. In select experiments, 1% CSE was added to cells as a positive control (Lewis et al., 2017; Sanders et al., 2017).

### 2.2 Real‐time cell invasion

Real‐time cell invasion was determined following the various treatments. An xCELLigence RTCA cell monitoring system was utilized to determine real time invasion of cells through adherence to the protocol suggested by the manufacturer (ACEA Biosciences, Blue Springs, MO, USA). Briefly, invasion was assessed in 16 well CIM‐Plates (n = 10 individual cell cultures per treatment; ACEA Biosciences, Blue Springs, MO, USA). The top wells were coated with a 1:40 matrigel concentration (Fisher Scientific, Pittsburg, PA) and cells were plated in the top chamber at a concentration of 20,000 cells/well in 2% FBS RPMI with total volume of 100 μL in the presence or absence of eCig liquid in the presence or absence of nicotine. The bottom chamber wells were filled with 160 μL of 10% FBS RPMI. The cells were then placed in the xCELLigence RTCA instrument and invasion readings were completed every 15 min for 24 hr. Invasive cells were obtained and expressed as cell invasion index.

### 2.3 Immunofluorescence

Immunofluorescence (IF) was performed to determine the localization of RAGE in treated and untreated cells. Slides were incubated overnight with antibody against RAGE (Cell Signaling Technology, Danvers, MA, USA) or an immunoglobulin G1‐negative control (Jackson Laboratories, West Grove, PA). Slides were incubated with a Texas red‐conjugated secondary for 1 hr and then 4,6‐diamidino‐2‐phenylindole dihydrochloride (DAPI) for nuclear counterstaining prior to mounting with glass coverslips. Slides were viewed with the appropriate excitation and emission rhodamine filter.

### 2.4 Periodontal inflammatory cytokine assessment

Medium from treated and control cells were screened using the multiplex Human Cytokine 17‐plex Magnetic Bead Kit (EMD Millipore, Billerica, MA). The Milliplex assay was performed as previously described (Kong & Krishnan‐Sarin, 2017). Beads and the appropriate detection antibodies are included in the Milliplex Multiplex Assay kit and were added to the control or treated conditioned media. The samples were incubated with antibody‐conjugated magnetic beads overnight at 4°C. Bead‐complexes were then read on a Magpix multiplex platform (Luminex Corporation, Austin, TX). Median fluorescent values were recorded from a minimum of 80 beads used in the data analysis. Standard curves and data analysis were performed using Milliplex Analyst 5.1 software (Millipore Corporation, Billerica, MA).

### 2.5 Statistical analysis

Results were checked for normality and data were shown as means ± SE. Differences in cell invasion and cytokine protein expression were determined between control and treated cells. Mann–Whitney tests were used to compare changes among the cytokines and for differences in invasion indexes. Significant differences between groups were noted at *p* < 0.05. Statistical analysis was completed with GraphPad Prism 7.0 software.

## 3 RESULTS

### 3.1 eCig liquid and OSCC invasion

**FIGURE 1:**
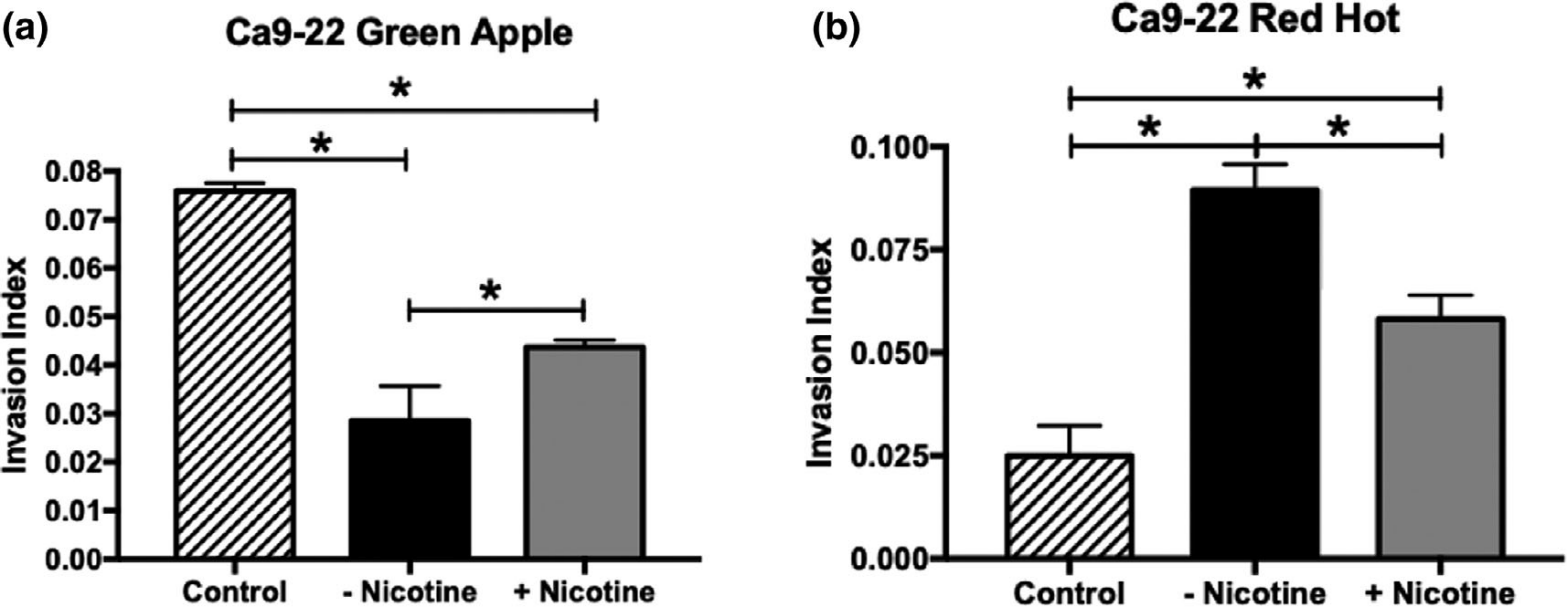
Ca9‐22 invasion with exposure to Green Apple or Red Hot eCig liquid. The addition of 2% Green Apple eCig liquid (a) significantly reduced cell invasion in the Ca9‐22 OSCC cells. This reduction in invasiveness significantly improved with the addition of 6 mg nicotine. The addition of 2% Red Hot significantly increased invasion of Ca9‐22 cells (b). Increased invasion induced by the flavoring alone was improved when 6 mg nicotine was included in the Red Hot flavoring (b). Invasion experiments were conducted with an n = 10 as outlined in the Methods section and statistically different values are noted as *p < .05

We first investigated the effects of eCig liquid on OSCC cell invasion. We observed that the addition of 2% Green Apple eCig liquid significantly reduced cell invasion (2.7‐fold; *p* < 0.0002) in Ca9‐22 cells (Figure 1a). The addition of nicotine to the media resulted in some improvement (35%, 1.7‐fold; p < 0.0002) despite the overall decrease in invasion observed in these cells when treated with 2% Green Apple liquid alone (Figure 1a). In contrast, treatment of Ca9‐22cells with 2% Red Hot significantly increased invasion (3.6‐fold; *p* < 0.0002). Interestingly, increased invasion mediated by Red Hot was decreased (30%, 2.3‐fold; p < 0.0002) with the inclusion of nicotine to the 2% Red Hot eCig liquid (Figure 1b).

**FIGURE 2:**
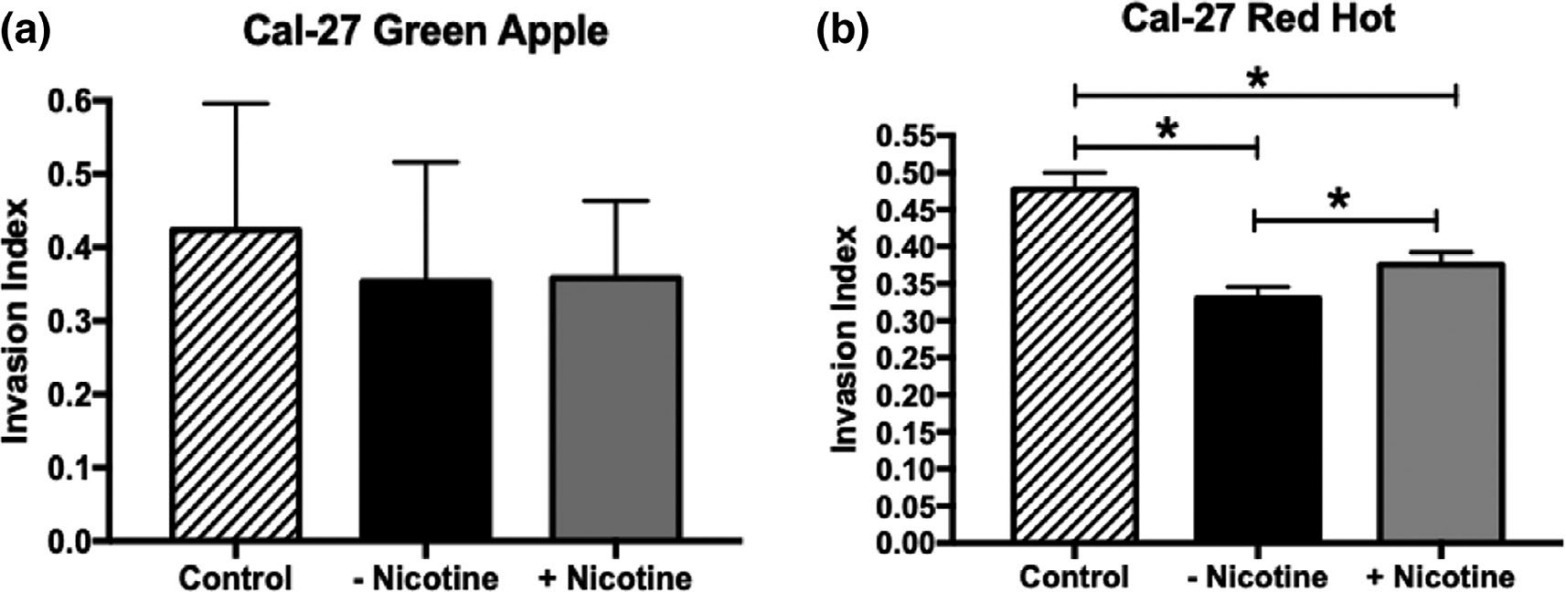
CAL‐27 invasion with exposure Green Apple or Red Hot eCig liquid. Green Apple eCig liquid did not affect CAL‐27 cell invasion regardless of the presence or absence of nicotine (a). Red Hot eCig liquid reduced CAL‐27 invasion which was mildly rescued with the addition nicotine (b). Invasion experiments were conducted in triplicate as outlined in the Methods section and statistically different values are noted as *p < .05

Treatment of CAL‐27 tongue squamous cells with 2% Green Apple did not affect cell invasion in either the presence or absence of nicotine (Figure 2a). Unlike 2% Green Apple, we observed decreased cell invasion (1.44‐fold; *p* < 0.002) in the CAL‐27 tongue squamous cells when treated with 2% Red Hot eCig liquid (Figure 2b). Decreased invasion was modestly reduced (7%,1.3‐fold; *p* < 0.002) when nicotine was added to the 2% Red Hot treatment (Figure 2b).

### 3.2 RAGE and cytokine release

**FIGURE 3:**
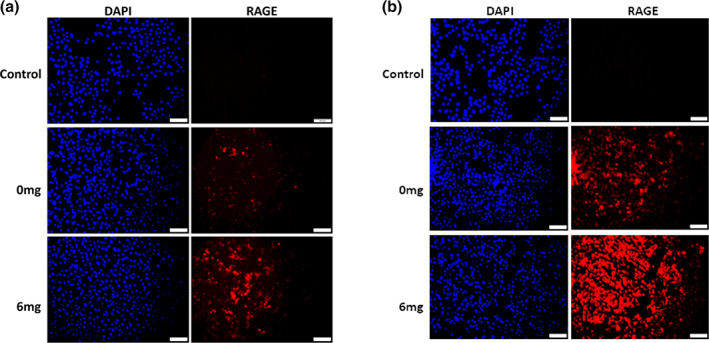
RAGE expression by Ca9‐22 cells treated with eCig liquid. Cells exposed to Green Apple (a) or Red Hot (b) both increased RAGE expression. Increased RAGE expression was further enhanced when nicotine was included with the eCig flavoring. Experiments were conducted in triplicate and Images (200x original magnification) are representative of the conducted cell culture experiments. Scale bars represent 100 m

**FIGURE 4:**
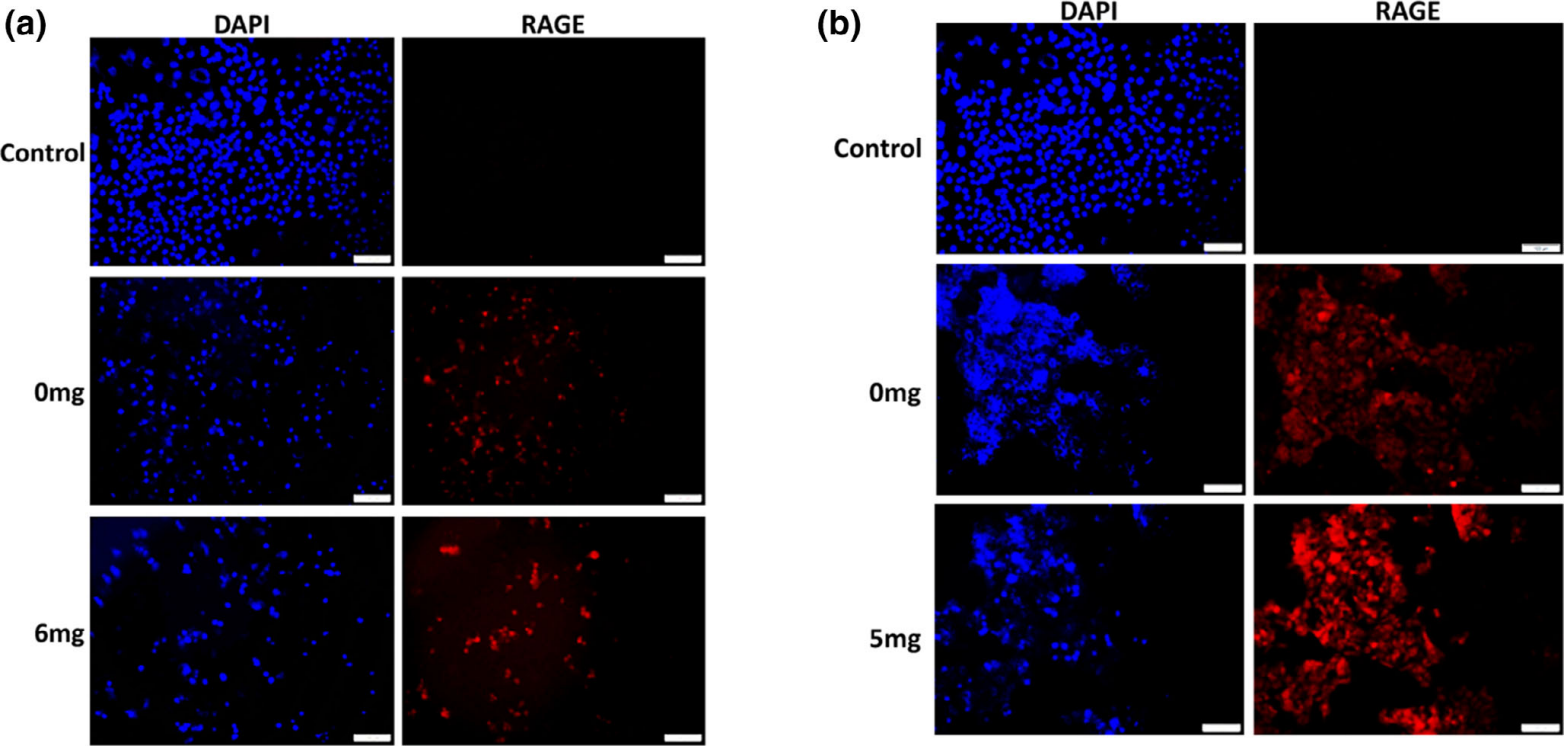
RAGE expression by CAL‐27 cells treated with in eCig liquid. Cells did not express RAGE in the absence of flavoring. Cells exposed to Green Apple (a) induced a modest up‐regulation of RAGE expression and CAL‐27 cells exposed to Green Apple liquid plus nicotine expressed slightly more RAGE when compared to cells exposed to eCig liquid alone. CAL‐27 cells exposed to Red Hot expressed high levels of RAGE with expression further increased when nicotine was added with the Red Hot eCig liquid (b). Experiments were conducted in triplicate and Images (200x original magnification) are representative of the conducted cell culture experiments. Scale bars represent 100 m

Previous studies by our lab showed a role for RAGE in mediating invasion and inflammation of OSCC cells when exposed to smoke environments (Chapman et al., 2018; Sanders et al., 2017). We therefore investigated the expression of RAGE during eCig liquid treatment of Ca9‐22 and CAL‐27 carcinoma cells. Immunofluorescence demonstrated increased RAGE staining in Ca9‐22 OSCC cells treated with 2% Green Apple or 2% Red Hot (Figure 3a,b). This increase in RAGE expression was potentiated when nicotine was added to either eCig liquid (Figure 3a,b). Similarly, RAGE staining was qualitatively increased following treatment of CAL‐27 cells that was further potentiated when nicotine was added (Figure 4a,b).

**FIGURE 5:**
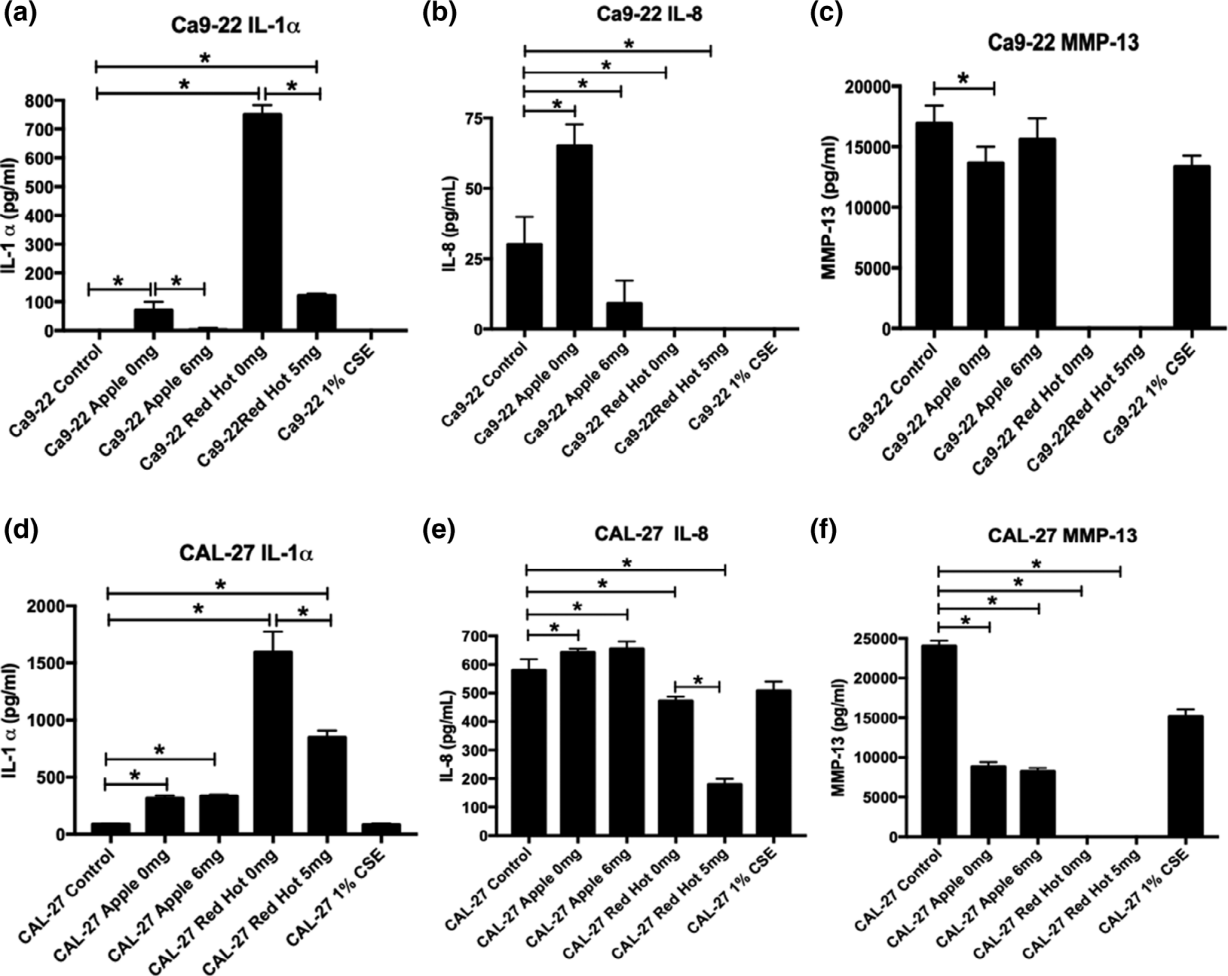
Expression of IL‐1α, IL‐8, and MMP‐13 by Ca9‐22 and CAL‐27 cells treated with eCig liquid. Ca9‐22 (a–c) and CAL‐27 (d–f) cells were treated as indicated and conditioned cell culture media was screened for IL‐1α(a and d), IL‐8 (b and e), and MMP‐13 (c and f). Secreted protein concentrations were obtained in quadruplicate and statistically different values are noted as *p < .05

Prior studies implicate RAGE signaling in the modulation of inflammation in response to exposure to cigarette smoke extract (CSE) via increased cytokine elaboration (Sanders et al., 2017). To determine to what extent cytokine release was regulated by eCig treatment, we performed protein assays that simultaneously screened key inflammatory cytokines observed during periodontal disease and compared expression to cells exposed to CSE as a positive control (Lewis et al., 2017; Sanders et al., 2017). The concentration of IFN‐γ, IL‐1β, IL‐12, IL‐4, IL‐6, IL‐10, IL‐12, IL‐17, MIP1‐α, MMP‐9, Osteoprotegerin, Osteopontin, Osteoactivin, RANK, TGFβ1, and TNF‐α released into cell culture media was below the levels of detection; thus, we were not able to accurately determine their concentrations in the media from either control or treated cells. In the Ca9‐22 cells, IL‐1α was significantly increased (54‐fold; *p* < 0.02) when cells were treated with 2% Green Apple when compared to controls (Figure 5a). The addition of nicotine to Green Apple treatment reduced the expression of this cytokine by 93% (3.4‐fold increase; *p* < 0.02, Figure 5a). Treatment of Ca9‐22 with 2% Red Hot demonstrated a very significant increase (580‐fold; p < 0.02) of IL‐1α when compared to controls (Figure 5a). When nicotine was added to the 2% Red Hot media, the increased IL‐1α was reduced by 95% (28‐fold; *p* < 0.02, Figure 5a). IL‐8 secretion was increased (2.2‐fold; *p* < 0.002) by treating Ca9‐22 cells with 2% Green apple alone (Figure 5b). In contrast, when nicotine was added to the cells in 2% Green Apple, IL‐8 was decreased (3.3‐fold; *p* < 0.002) when compared to controls (Figure 5b). Interestingly IL‐8 secretion was below detection in the 2% Red Hot treated Ca9‐22 cells (Figure 5b). MMP‐13 levels were decreased by 2% Green Apple treatment (1.2‐fold increase; *p* < 0.03) in Ca9‐22 cells (Figure 5c). When nicotine was added, this small decrease was reversed to basal levels observed in controls (Figure 5c).

Treatment of the CAL‐27 tongue squamous cells with 2% Green Apple showed increased IL‐1α (3.6‐fold; *p* < 0.02) when compared to controls (Figure 5d). The addition of nicotine to the 2% Green Apple did not affect IL‐1α secretion levels by these cells (3.8‐fold; *p* < 0.02). Similarly, treatment of CAL‐27 cells with 2% Red Hot alone increased IL‐1α (17.8‐fold; *p* < 0.02) when compared to controls (Figure 5d). Interestingly, addition of nicotine reduced IL‐1α levels by 52% (8.5‐fold increase; *p* < 0.02) compared to the treatment of 2% Red Hot alone (Figure 5d). IL‐8 levels were mildly increased (1.1‐fold; *p* < 0.04) in CAL‐27 cells that were treated with 2% Green Apple and this increase was not affected when nicotine was added (Figure 5e). When CAL‐27 cells were treated with 2% Red Hot, IL‐8 levels were decreased (1.2‐fold; *p* < 0.04) when compared to control cells (Figure 5e). This decrease was potentiated (3.2‐fold; *p* < 0.04) by the addition of nicotine (Figure 5e). MMP‐13 levels were reduced when CAL‐27 cells were treated with 2% Green Apple in the absence (2.7‐fold; *p* < 0.0001) or presence (2.9‐fold; *p* < 0.001) of nicotine (Figure 5f). Interestingly, MMP‐13 secretion was below the detection limit in the 2% Red Hot treated CAL‐27 cells (Figure 5f).

## 4 DISCUSSION

Cigarette smoke is among the top 10 contributors to the worldwide health burden; however, eCigs are a phenomenon that has emerged in the United States only during the last decade (Dai & Leventhal, 2019). Flavored eCig vaping is steadily rising. Troubling trends for eCig use suggest rapid increases among several population demographics and an astonishing 1 out of 10 current adolescent users (Kong & Krishnan‐Sarin, 2017; Singh et al., 2016). While attempts to regulate eCig use are underway (Food and Drug Administration, HHS, 2016), the World Health Organization has stated eCig vaping should not be recommended until its true toxicity profile and potential ill health effects have been properly vetted (Food and Drug Administration, HHS, 2016). Such adverse health effects associated with eCig use include bronchitis, mouth/throat irritation, headaches, nausea, airway obstruction, bronchospasm, inflammation, and cardiovascular effects (elevated heart rate, blood pressure, and vessel stiffness) (Aberle, Abtin, & Brown, 2013; Etter, 2010; Vakaliet al., 2013; Polosa et al., 2014; Vansickel, Cobb, Weaver, & Eissenberg, 2010; Vardavas et al., 2012; Wang et al., 2018). To determine the effects of eCig liquid and nicotine in OSCC progression, cell invasion was assessed in commonly studied OSCC cell lines. We measured invasion during exposure to two popular eCig liquids: Cinnamon Red Hots (Red Hot) or Reds Apple Juice (Green Apple) in the presence or absence of nicotine. In the Ca9‐22 cells, we observed that invasion was increased when cells were treated with Red Hot alone. In contrast, cell invasion was decreased when cells were treated with Green Apple alone. These results suggest a flavor dependent effect in the regulation of cell invasion. As many users of eCigs seek an alternative means of nicotine exposure aside from traditional smoking, an understanding of nicotine related effects is essential. The addition of nicotine in the presence of Red Hot diminished the increased invasion observed in exposed cells. This was unexpected as these results suggest that perhaps the addition of nicotine exerted measurable protection of invasion in Ca9‐22 cells treated with Red Hot. When nicotine was added to the Green Apple Ca9‐22 treated cells, we observed a near reversal in the invasion index. Although these nicotine results were not capable of reversing invasion to basal levels, these data suggest that nicotine is a key variable of eCig use that must be further explored. Furthermore, our research reveals the plausibility that constituents in the eCig liquid may be in part responsible for reduced invasion observed when nicotine is present (Sun & Ma, 2015). Together these data suggest that different molecular means are plausibly involved in the regulation of these cancerous cells that are dependent on flavoring and the presence of nicotine.

In terms of the CAL‐27 cells, we did not detect any effect in cell invasion when cells were treated with Green Apple in the presence or absence of nicotine. This was an unexpected difference compared to our observations of the Ca9‐22 cells and perhaps this discovery is related to the previous observation that CAL‐27 cells are tongue squamous cell carcinoma cells known to have non‐typical OSCC behaviors (Jiang et al., 2009). This alternative response compared to Ca9‐22 cells was also observed when Red Hot was added to the CAL‐27 cells. In this instance, invasion was decreased in the presence of eCig liquid alone and significantly recovered when nicotine was added to the cells. As such, alternative effects may characterize different flavorings and the concept that cellular response coordination is cell‐dependent cannot be discounted.

Previous studies in our lab has linked RAGE expression to both inflammation and the regulation of invasion during exposure of OSCC to smoke environments (Chapman et al., 2018; Sanders et al., 2017). We observed detectible RAGE protein expression in both cell types via immunofluorescence staining. Interestingly we observed increased staining for RAGE with both eCig liquids in Ca9‐22. This increase was further potentiated when nicotine was added to the treatment. These data suggest the likelihood that RAGE correlates to pathways involved in the regulation of invasion in Ca9‐22 cells, although additional research is necessary to identify direct influence of these outcomes. Similar results were observed for the CAL‐27 tongue cells. These results collectively suggest that in both cell types, RAGE may be involved in alternative cell invasion indexes, dependent on the cell type, during eCig treatment (Chapman et al., 2018; Sanders et al., 2017). We anticipate performing follow up studies that target RAGE in order to elucidate to what extent RAGE availability modulates invasiveness. RAGE signaling generally involves the activation of several MAP kinases and the activation of NF‐κB (Schmidt & Stern, 2001). To further determine RAGE involvement in these cells, investigations must also be conducted that assess these signaling intermediates as well as nuclear activation of NF‐κB in treated cells as well as controls.

We detected increased inflammatory cytokines such as IL‐1α and IL‐8 when Ca9‐22 were treated with either of the eCig liquids. IL‐1α and IL‐1β are products of distinct genes but are similar in their modulation of inflammation. Both bind the same receptor and are upregulated during an inflammatory response. IL‐8 is a heparin binding member of the alpha, or CXC family of chemokines. In addition of pro‐inflammatory effects, IL‐8 oligomerizes and binds G‐protein coupled receptors to enhance angiogenesis during pathologies including cancer. The observed increases in both IL‐1α and IL‐8 were reduced when nicotine was added in these cells. HoweverMMP‐13 was decreased with Apple Green treatment and not detectable in Red Hot treated Ca9‐22 cells. MMPs, including MMP‐13, are zinc and calcium dependent endopeptidases that degrade extracellular matrix. MMP‐13 specifically has been shown to degrade aggrecans and diverse collagens while functioning during cell‐matrix interactions. Cell‐matrix interactions are key during invasion. Together with the RAGE immunofluorescence, differential cytokine elaboration suggests that RAGE expression may be involved in the development of inflammation and less so during the regulation of invasion in Ca9‐22 cells. Importantly, nicotine treatment seemed to have a marginally protective role for inflammation created by both eCig liquids. This protective role of nicotine has been observed in other diseases and it is suggested to be orchestrated by regulating cytokine expression (Lakhan & Kirchgessner, 2011; Piao et al., 2009). To our knowledge, this protection has not previously been shown during OSCC progression in humans. In the CAL‐27 cells, we observed increased IL‐1α when cells were treated with both eCig liquids. Interestingly, nicotine did not affect the levels of this cytokine in the Green Apple treated cells. In contrast a significant decrease of IL‐1α was observed in cells treated with Red Hot. These results suggest a divergent signaling pathway may be initiated in these cells in a flavor dependent manner; however, RAGE augmentation still suggests RAGE participation. Furthermore, it seems that nicotine protection is also flavor dependent in the CAL‐27 cells. IL‐8 was mildly increased during Green Apple treatment and was not affected by nicotine addition. In contrast, Red Hot induced a significant increase of IL‐8 that was not reversed by the addition of nicotine. MMP‐13 was decreased in these cells treated with Green Apple and below the detection limit when cells were treated with Red Hot.

In general, our experiments suggest that different eCig flavors alternatively affect OSCCs, making it difficult to determine specific effects and/or consequences of eCig use. We demonstrate the plausibility that RAGE availability correlates with eCig‐mediated invasion and inflammation. Additional studies are critically needed to further identify the relationship between RAGE signaling and the pathological behavior of OSCC cells during exposure. Such studies should include RAGE targeting and downstream effects in terms of both signaling intermediates and functional molecule elaboration. These additional experiments should also be conducted in cells exposed to varying levels of nicotine in order to contextualize combinatorial effects of nicotine and popular eCig flavorings. Lacking would also be a more exhaustive screening of effects in additional cell lines and primary OSCC cells as some cell lines, possibly including Ca9‐22 cells, may be problematic (Zhao et al., 2011). Accordingly, the two cell lines used in the current work were derived from the male and a complete assessment would consider cells from both males and females over a wide range of ages. Such future undertaking would clarify the molecular underpinning of exposing OSCC cells to vaporized eCig liquids and resulting invasiveness and inflammatory signaling.

## CONFLICT OF INTEREST

The authors report no conflict of interest.

## ETHICS STATEMENT

Ethics committee/institute approval was not required because established cell lines were used for these experiments.
